# Muscle fitness and physical function in children and adolescents with newly diagnosed cancer

**DOI:** 10.3389/fped.2026.1769860

**Published:** 2026-05-28

**Authors:** Peter Schmidt-Andersen, Hanne Bækgaard Larsen, Anna Pouplier, Martin Bjørn Hansen, Liv Andres-Jensen, Sine Lykkedegn, Henrik Hasle, Klaus G. Müller, Jan Christensen, Martin K. Fridh

**Affiliations:** 1 Department of Pediatrics and Adolescent Medicine, Copenhagen University Hospital—Rigshospitalet, Copenhagen, Denmark; 2 Department of Occupational Therapy and Physiotherapy, Copenhagen University Hospital—Rigshospitalet, Copenhagen, Denmark; 3 Institute of Clinical Medicine, University of Copenhagen, Copenhagen, Denmark; 4 Department of Pediatric Hematology and Oncology, H.C. Andersen Children’s Hospital, Odense University Hospital, Odense, Denmark; 5 Department of Pediatrics and Adolescent Medicine, Aarhus University Hospital, Aarhus, Denmark; 6 Department of Public Health, Section of Social Medicine, Faculty of Health and Medical Sciences, University of Copenhagen, Copenhagen, Denmark

**Keywords:** cancer treatment–related deconditioning, cross-sectional study, early rehabilitation, muscle strength and endurance, pediatric exercise oncology, physical capacity, physical capacity assessment

## Abstract

**Background and aims:**

Maintaining muscle health is crucial for children undergoing cancer treatment. However, the extent of impairments during the early stages of treatment remains sparsely investigated. Therefore, we investigated muscle fitness—muscle strength, muscle power, and muscle endurance—and physical function in children with newly diagnosed cancer compared to community controls.

**Methods:**

We compared parameters of muscle strength (assessed by isometric knee-extension, bench press and handgrip strength), muscle power and physical function [by countermovement jump and timed-up-and-go (TUG)], and lower body strength and muscle endurance [by 30 and 60-second sit-to-stand test (STS)] between children with newly diagnosed cancer (age 6–17.9 years) and community controls. Children with cancer were assessed within 31 days of treatment initiation.

**Results:**

We included 123 children with cancer (58% boys; median age 12 years, IQR 8–15), assessed within a median of 9 (IQR 6–14) days after cancer treatment initiation, and 221 community controls (61% boys; median age 12 years, IQR 9–14). All outcomes were significantly lower in children with cancer compared with community controls except handgrip strength. Knee extension strength was 15.8% lower (mean diff.: −5.46 kg, 95% CI: −9.98; −0.93, *p* = 0.02), bench press 33.1% lower (–10.09 kg, 95% CI: −15.18; −4.99, *p* = <0.01), and handgrip 11.7% lower (–2.57 kg, 95% CI: −5.25; 0.11, *p* = 0.06). Countermovement jump was reduced by 40.6% (–9.19 cm, 95% CI: −12.39; −5.99, *p* = <0.01). Lower body strength was reduced in the 30 s STS (–6.04 repetitions, 95% CI: −7.45; −4.62, *p* < 0.01), and muscle endurance was similarly reduced in the 60 s STS (–13.54 repetitions, 95% CI: −16.31; −10.77, *p* < 0.01). In addition, functional impairments were observed, with a 29.8% slower TUG (1.18 s, 95% CI: 0.79; 1.57, *p* < 0.01). These deficits represent substantial impairments in muscle fitness and physical function, consistent with difficulties performing everyday functional tasks.

**Conclusions:**

Children with cancer have impaired muscle fitness and physical performance within the first month of treatment. Our results suggest that rehabilitation should be implemented early during treatment to counteract further loss of muscle strength, muscle power, and muscle endurance, with the potential to support motor development and maintain sense of normality.

## 1 Introduction

Maintaining muscle health is crucial for children undergoing cancer treatment (1), yet the extent and timing of muscle fitness impairments in the early treatment phases remain sparsely investigated (2).

Cancer treatment in children negatively affects muscle fitness—including muscle mass, strength, power, and endurance (3). These components are essential for motor development and maintaining functional mobility throughout childhood (1, 4). Beyond bodily movement, muscle fitness plays a crucial role in whole-body homeostasis and thereby metabolism and immune function (1, 5). During therapy, children frequently experience physical impairments associated with treatment-related side effects such as peripheral neuropathy, altered body composition, and reduced physical functioning (6–8). Although some recovery occurs post-treatment, long-term complications remain prevalent; approximately 80% of adult survivors of childhood cancer report musculoskeletal complications, and over 15% experience severe muscle weakness (9–13). Additionally, survivors of childhood cancer have a ∼35% higher risk of skeletal adverse events within the first year after completing treatment compared with the general population (14). These impairments contribute to persistent fatigue, reduced independence in daily activities, and limited participation in social life—ultimately compromising quality of life and significantly increasing the risk of severe chronic health conditions (11, 15–18).

Although the long-term effects of cancer treatment on muscle fitness are well recognized, the onset and magnitude of impairments during the early treatment phase remain insufficiently characterized (2). Existing studies are limited by small sample sizes, heterogeneous populations, and inconsistent outcome measures, resulting in low certainty of evidence (2). This knowledge gap limits our ability to design timely, targeted rehabilitation strategies aimed at preserving muscle function from the initiation of cancer treatment.

To inform early intervention strategies in childhood cancer, it is essential to determine the extent to which, and at what points during the treatment trajectory, muscle fitness is impaired.

Accordingly, this cross-sectional study aims to assess muscle fitness—specifically muscle strength, muscle power, and muscle endurance—and physical function within the first month of cancer treatment in children and adolescents, compared with community controls. Additionally, the study explores associations between muscle fitness impairments and treatment duration.

## 2 Materials and methods

### 2.1 Design and setting

For this cross-sectional study, baseline data from the randomized controlled trial, Integrative neuromuscular training in adolescents and children treated for cancer—INTERACT (Clinical Trial registration NCT04706676), were included. Children were included between January 2021 and May 2025, from three hospitals in Denmark: Copenhagen University Hospital‒Rigshospitalet, Odense University Hospital, and Aarhus University Hospital. Furthermore, a community control group of 221 children and adolescents without a history of cancer was included for comparison. These participants were enrolled from the Acute Lymphoblastic Leukemia Survivor Toxicity and Rehabilitation (ALL-STAR) cohort (19) from January 2019 to June 2023.

### 2.2 Participants

The study included children newly diagnosed with cancer or cancer relapse, treated with chemotherapy and/or irradiation, and aged 6.0‒17.9 years at diagnosis. Children with mental illness or physical disability, terminal illness, and children unable to speak Danish were excluded (20). Children were included in the study within 14 days of treatment initiation, and physical assessments were conducted within one month of cancer treatment.

Community controls were recruited among friends and relatives of survivors of childhood acute lymphoblastic leukemia enrolled in the ALL-STAR cohort (19). Controls were selected within the same age range (6.0–17.9 years) as the cancer group but were not individually age- or sex-matched.

### 2.3 Outcomes

#### 2.3.1 Lower body strength—isometric knee extension

Isometric knee extension strength was assessed using a custom-built strength ergometer (Gym 2000®, Vikersund, Norway), equipped with a dynamometer (U2A100kg, Hottinger, Germany). The participant sat upright with hips and knees at 90° flexion and performed unilateral maximal isometric contractions for at least 5 s. Participants completed a minimum of three and up to five attempts, with a 1-minute rest between each. Additional trials were permitted only when the most recent score exceeded the previous one. The highest force (kg) was recorded.

#### 2.3.2 Upper body strength—isometric bench press

Isometric upper body strength was measured using the same ergometer setup as for knee extension. Participants lay supine with arms positioned at 150% of biacromial width and elbows flexed at 90°. After instructions, they were instructed to push upward with maximal force while maintaining position. Participants completed a minimum of three and up to five attempts, with a 1 min rest between each. Additional trials were permitted only when the most recent score exceeded the previous one. The highest force (kg) was recorded.

#### 2.3.3 Upper body strength—handgrip strength

Handgrip strength was measured using a hand-held dynamometer (Jamar, Patterson Medical, Illinois, USA) (21), in a seated position with the elbow at 90° flexion and the underarm resting at the armrest of the chair. Two maximal efforts were performed with each hand. The highest score (kg) was used.

#### 2.3.4 Muscle power—countermovement jump

Lower extremity muscle power was assessed in a sub-sample of participants as the equipment were only available at one study site (Rigshospitalet). Using a mobile force plate (FP4, HUR-Labs Oy, Tampere, Finland). Participants were instructed to perform a countermovement jump (knee angle to 90°) aiming for maximal vertical height with hands on hips and land on both feet. Participants completed a minimum of three and up to five attempts, with a 1 min rest between each. Additional trials were permitted only when the most recent score exceeded the previous one. The highest score (jump height in cm) was recorded.

#### 2.3.5 Lower extremity strength and endurance—30 and 60 s sit-to-stand test

Participants performed the 30 s and 1 min sit-to-stand test using a standard chair (22, 23) They were instructed to rise and sit repeatedly as many times as possible with arms crossed or at their sides. The number of repetitions at 30 s (strength) and 60 s (endurance) was recorded.

#### 2.3.6 Physical function—timed up and go test

Physical Function was measured with the Timed Up and Go (TUG) test (24). Seated in a standard chair, participants were instructed to rise, walk 3 meters, turn, return, and sit down as fast as possible. Time (seconds) was recorded to two decimals. Three attempts were conducted; the shortest time was recorded.

### 2.4 Statistical methods

All outcomes were analyzed using ANCOVA models to compare patients with community controls. Three models were fitted sequentially: (1) an unadjusted model, (2) a model adjusted for age and sex and their interaction, and (3) a model, further adjusted for diagnosis.

As a subgroup analysis, we examined (1) differences between each diagnostic subgroup [hematologic malignancies, a hematologic subgroup of children with acute lymphoblastic leukemia (ALL), extracranial solid tumors, and central nervous system (CNS) tumors] and community controls, and (2) differences between diagnostic subgroups within the patient cohort, using the same analytical approach.

Further, we analyzed the association between outcomes and time since initiation of cancer treatment in the patient group (pan-cancer) and within each diagnostic group, adjusting for sex, age, and diagnosis to assess whether longer exposure to treatment was associated with poorer outcomes.

As muscle power was only assessed in participants from Rigshospitalet, these results were included only in the pan-cancer analyses and not in the comparisons between diagnostic subgroups.

To assess comparability between the patient and control groups in terms of sex, age, and BMI, data were first checked for normality. Independent *t*-tests were used for age and BMI, and a chi-square test for sex distribution. Statistical significance was defined as *p* < 0.05, and results are presented as mean differences with 95% confidence intervals.

## 3 Results

### 3.1 Participant characteristics

**Figure 1:**
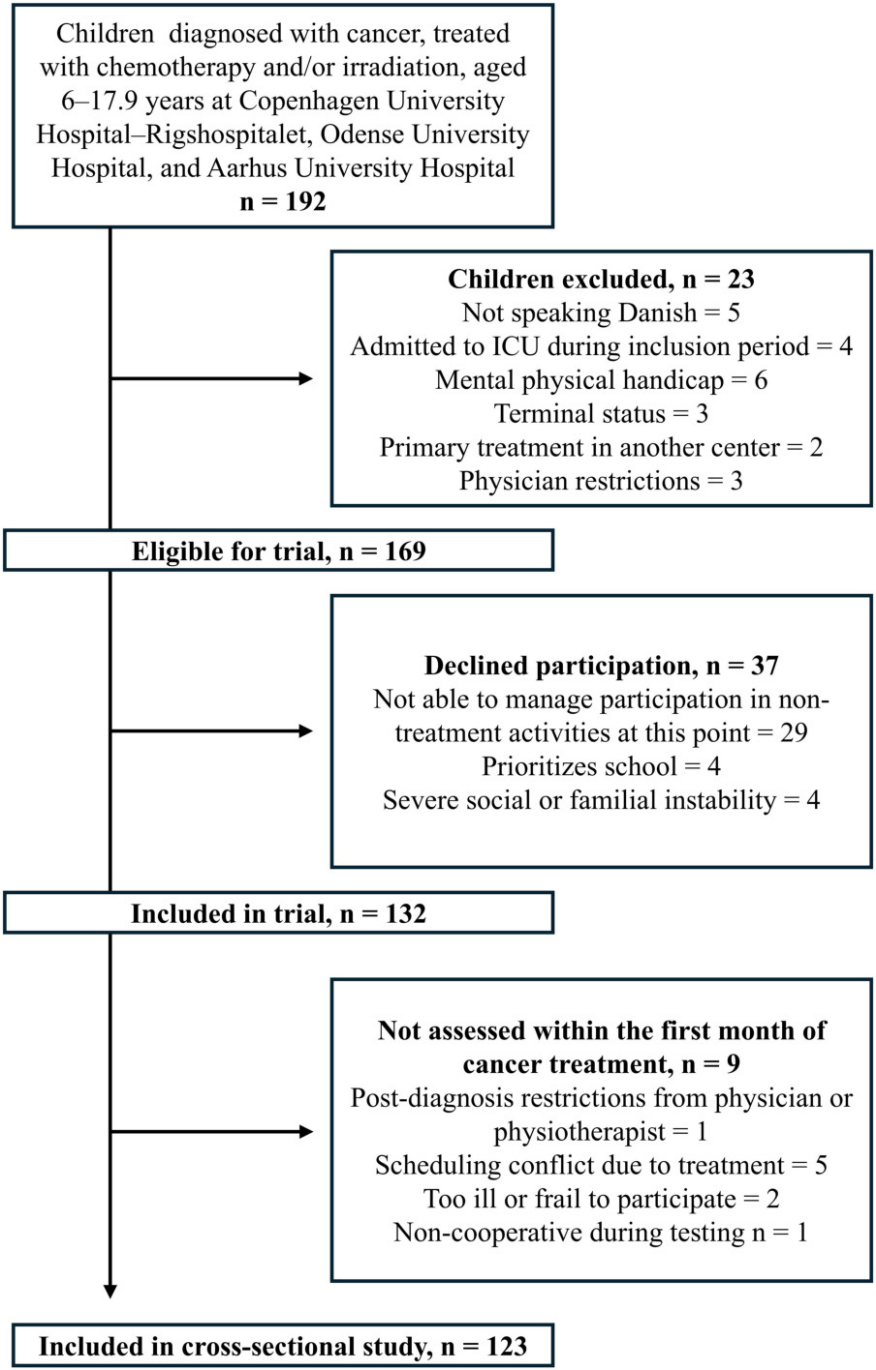
Flowchart. ICU, intensive care unit.

Between January 2021 and May 2025, 132 children out of 169 eligible (78%) were included in the INTERACT trial, of which 123 (73%) were assessed within the first month of cancer diagnosis and, therefore, included. The enrollment process is described in Figure 1.

A detailed description of anthropometric and physical characteristics of study participants, including community controls, is described in Table 1. We did not find any anthropometric differences in sex, age, or BMI between the two groups; hence, these were comparable within the timeframe for this cross-sectional study. Seven participants had a cancer relapse.

**Table 1:** Anthropometric and clinical characteristics.

A – Characteristics	Children with cancer (*n* = 123)	Community controls (*n* = 221)	*p*-value btw. groups
Sex (males/females)	71/52 (58% boys)	134/87 (61% boys)	0.58
Age (median years)	12 (IQR: 8.0–14.5)	12 (IQR: 9- 14)	0.99
BMI	18.20 (IQR: 15.19–20.09	17.65 (IQR: 16.18–20.12)	0.68
Days since diagnosis (Assessment timing)	9 (IQR: 6.0–14.0)		
Diagnosis			
Hematologic cancer	83 (68%)		
*– ALL*	*35 (29%)*		
*– Non-Hodgkin Lymphoma*	*22 (18%)*		
*– Hodgkin Lymphoma*	*16 (13%)*		
*– AML*	*7 (6%)*		
*– Other Lymphomas*	*3 (2%)*		
Extracranial solid tumors	31 (25%)		
*– Bone tumors*	13 (11%)		
*– Embryonal Tumors*	5 (4%)		
*– Germ Cell Tumors*	5 (4%)		
*– Soft Tissue Sarcomas*	3 (2%)		
*– Carcinomas*	2 (2%)		
*– Other Solid Tumors*	3 (2%)		
CNS tumors	9 (7%)		

B – Outcome measures	Children with cancer (*n* = 123)	Community controls (*n* = 221)	*p*-value btw. groups
Knee extension (kg)	29.08 ± 17.9	34.54 ± 14.07	0.018
Bench press (kg)	20.44 ± 16.2	30.53 ± 17.69	<0.001
Handgrip (kg)	19.31 ± 12.2	21.87 ± 11.93	0.06
CMJ (cm)	13.43 ± 6.3	22.62 ± 6.93	<0.001
Sit-to-stand 30 s (rep)	17.15 ± 5.59	23.19 ± 6.04	<0.001
Sit-to-stand 60 s (rep)	32.69 ± 11.09	46.23 ± 11.69	<0.001
Timed Up and Go (sec)	5.14 ± 2.76	3.96 ± 0.57	<0.001

Tables 1A,B: Anthropometric and clinical characteristics of participants—children with cancer and children without a history of cancer (community controls). Table 1B reports mean values across all outcomes, including standard deviation (± SD). ALL, acute lymphoblastic Leukemia. Please note that the subgroup of children with ALL is a sample within the hematologic cancer group. CNS, central nervous system; CMJ, countermovement jump.

### 3.2 Muscle fitness and physical function within the acute stage of cancer treatment

We found that general muscle fitness was significantly lower in children with cancer compared with community controls, in all assessed parameters except for handgrip strength, as shown in Table 2. Specifically, we found a 15.8% isometric knee extension strength in children with cancer compared with community controls (mean diff.: −5.46 kg, 95% CI: −9.98 to −0.93, *p* = 0.02). Bench press strength was 33.1% lower (mean diff.: −10.09 kg, 95% CI: −15.18 to −4.99, *p* < 0.01). Countermovement jump performance was 40.6% lower in children with cancer (mean diff.: −9.19 cm, 95% CI: −12.39 to −5.99, *p* < 0.01). Furthermore, children with cancer had a 26.0% lower performance in the 30-second Sit-to-Stand test (mean diff.: −6.04, 95% CI: −7.45 to −4.62, *p* < 0.01), and a 29.3% lower performance in the 60 s Sit-to-Stand test (mean diff.: −13.54, 95% CI: −16.31 to −10.77, *p* < 0.01).In addition, children with cancer were 29.8% slower in the Timed Up and Go test (mean diff.: 1.18 s, 95% CI: 0.79 to 1.57, *p* < 0.01).These findings remained robust— and showed further decline— when adjusting for age, sex, and diagnosis in all parameters except for physical function measured by the Timed Up and Go test (Table 2).

**Table 2:** Comparison of muscle fitness and physical function.

Knee extension, Bench press, hand grip strength, countermovement jump, Timed Up and Go test, Sit-to-Stand (30 and 60 s): comparison between children with newly diagnosed cancer (within one month of cancer treatment) and community controls						
Outcome	Unadjusted mean diff (95% CI)	*p*-value	Age*Sex adjusted mean diff (95% CI)	*p*-value	Age*Sex + diagnosis adjusted mean diff (95% CI)	*p*-value
Knee extension (kg), *n* = 111	−5.46 [−9.98 to −0.93]	0.02	−5.38 [−8.29 to −2.47]	<0.01	−7.00 [−15.62 to 1.61]	0.11
Bench press (kg), *n* = 81	−10.09 [−15.18 to −4.99]	<0.01	−11.21 [−14.85 to −7.56]	<0.01	−13.30 [−23.44 to −3.16]	0.01
Handgrip (kg) *n* = 122	−2.57 [−5.25 to 0.11]	0.06	−2.54 [−4.01 to −1.07]	<0.01	−2.27 [−6.45 to 1.91]	0.29
Countermovement jump (cm), *n* = 24	−9.19 [−12.39 to −5.99]	<0.01	−8.8 [−11.13 to −6.47]	<0.01	−15.92 [−25.89 to −5.95]	<0.01
Sit-to-stand 30 s (repetitions), *n* = 98	−6.04 [−7.45 to −4.62]	<0.01	−5.94 [−7.33 to −4.55]	<0.01	−8.23 [−12.10 to −4.35]	<0.01
Sit-to-stand 60 s (repetitions), *n* = 97	−13.54 [−16.31 to −10.77]	<0.01	−13.37 [−16.09 to −10.65]	<0.01	−16.00 [−23.56 to −8.44]	<0.01
Timed Up and Go (sec), *n* = 114	1.18 [0.79 to 1.57]	<0.01	1.15 [0.77 to 1.53]	<0.01	1.34 [ 0.29 to 2.39]	0.01

Table 2: comparison of muscle fitness and physical function between children newly diagnosed with cancer and community controls. CI, Confidence intervals. The analysis comprises three models: (a) unadjusted; (b) adjusted for age, sex, and their interaction; (c) adjusted for age, sex, their interaction, and diagnosis.

### 3.3 Muscle fitness and physical function within the acute stage of cancer treatment in different diagnostic groups compared to community controls

Children with cancer, across diagnostic groups, generally showed lower muscle fitness and functional performance compared with community controls; detailed results by diagnostic group are presented in Tables 3A–C.

**Table 3:** Comparison of muscle fitness and physical function in different diagnostic groups.

A Knee extension, bench press, hand grip strength, timed up and go test, sit-to-stand (30 and 60 s): comparison between children with hematologic cancers and community controls				
Outcome	Unadjusted mean diff (95% CI)	*p*-value	Age*Sex adjusted mean diff (95% CI)	*p*-value
Knee extension (kg), *n* = 78	−7.44 [−12.20 to −2.68]	<0.01	−6.83 [−9.84 to −3.83]	<0.01
Bench press (kg), *n* = 53	−9.98 [−15.90 to −4.06]	<0.01	−11.71 [−15.89 to −7.53]	<0.01
Handgrip (kg) *n* = 81	−4.28 [−7.27 to −1.28]	<0.01	−3.49 [−5.14 to −1.84]	<0.01
Sit-to-stand 30s (repetitions), *n* = 67	−6.31 [−7.98 to −4.64]	<0.01	−6.25 [−7.88 to −4.63]	<0.01
Sit-to-stand 60s (repetitions), *n* = 66	−14.32 [−17.58 to −11.06]	<0.01	−14.21 [−17.40 to −11.02]	<0.01
Timed Up and Go (sec), *n* = 79	0.87 [0.63 to 1.11]	<0.01	0.87 [0.63 to 1.10]	<0.01
A1 Knee extension, Bench press, hand grip strength, Timed Up and Go test, Sit-to-Stand (30 and 60 s): comparison between children with acute lymphoblastic leukemia and community controls				
Knee extension (kg), *n* = 34	−14.73 [−20.03 to −9.44]	<0.01	−11.07 [−14.33 to −7.81]	<0.01
Bench press (kg), *n* = 24	−16.74 [−24.07 to −9.42]	<0.01	−14.63 [−19.58 to −9.68]	<0.01
Handgrip (kg) *n* = 34	−7.32 [−11.49 to −3.16]	<0.01	−3.42 [−5.75 to −1.08]	<0.01
Sit-to-stand 30s (repetitions), *n* = 27	−7.64 [−10.07 to −5.20]	<0.01	−7.83 [−10.23 to −5.43]	<0.01
Sit-to-stand 60s (repetitions), *n* = 26	−18.31 [−23.04 to −13.58]	<0.01	−18.55 [−23.21 to −13.90]	<0.01
Timed Up and Go (sec), *n* = 33	1.16 [0.88 to 1.45]	<0.01	1.14 [0.85 to 1.43]	<0.01
B Knee extension, Bench press, hand grip strength, Timed Up and Go test, Sit-to-Stand (30 and 60 s): comparison between children with extracranial solid tumor and community controls				
Knee extension (kg), *n* = 27	0.98 [−5.77 to 7.72]	0.77	−0.69 [−4.99 to 3.61]	0.75
Bench press (kg), *n* = 22	−9.26 [−17.43 to −1.09]	0.03	−9.73 [−15.12 to −4.33]	<0.01
Handgrip (kg) *n* = 31	1.13 [−3.49 to 5.74]	0.63	−0.25 [−2.77 to 2.26]	0.84
Sit-to-stand 30s (repetitions), *n* = 22	−4.37 [−6.98 to −1.77]	<0.01	−4.05 [−6.62 to −1.48]	<0.01
Sit-to-stand 60s (repetitions), *n* = 22	−10.37 [−15.43 to −5.30]	<0.01	−9.73 [−14.71 to −4.74]	<0.01
Timed Up and Go (sec), *n* = 25	2.12 [1.39 to 2.86]	<0.01	1.94 [1.21 to 2.67]	<0.01
C Knee extension, Bench press, hand grip strength, Timed Up and Go test, Sit-to-Stand (30 and 60 s): comparison between children with CNS tumors and community controls				
Knee extension (kg), *n* = 6	−8.63 [−20.19 to 2.92]	0.14	−7.12 [−14.17 to −0.08]	0.05
Bench press (kg), *n* = 6	−14.07 [−28.59 to 0.44]	0.06	−13.16 [−22.20 to −4.13]	<0.01
Handgrip (kg) *n* = 9	−0.17 [−7.83 to 7.48]	0.96	−2.27 [−6.34 to 1.80]	0.27
Sit-to-stand 30s (repetitions), *n* = 9	−8.08 [−12.07 to −4.08]	<0.01	−8.26 [−12.20 to −4.32]	<0.01
Sit-to-stand 60s (repetitions), *n* = 9	−15.57 [−23.31 to −7.83]	<0.01	−15.97 [−23.59 to −8.35]	<0.01
Timed Up and Go (sec), *n* = 9	+1.23 [0.85 to 1.61]	<0.01	+1.29 [0.91 to 1.66]	<0.01

Table 3A–C: Comparison of Muscle fitness and physical function within the acute stage of cancer treatment in different diagnostic groups. Please note that the subgroup of children with ALL(A1) is a sample within the hematologic cancer group.

#### 3.3.1 Differences in muscle fitness and physical function between diagnostic groups

**Figure 2:**
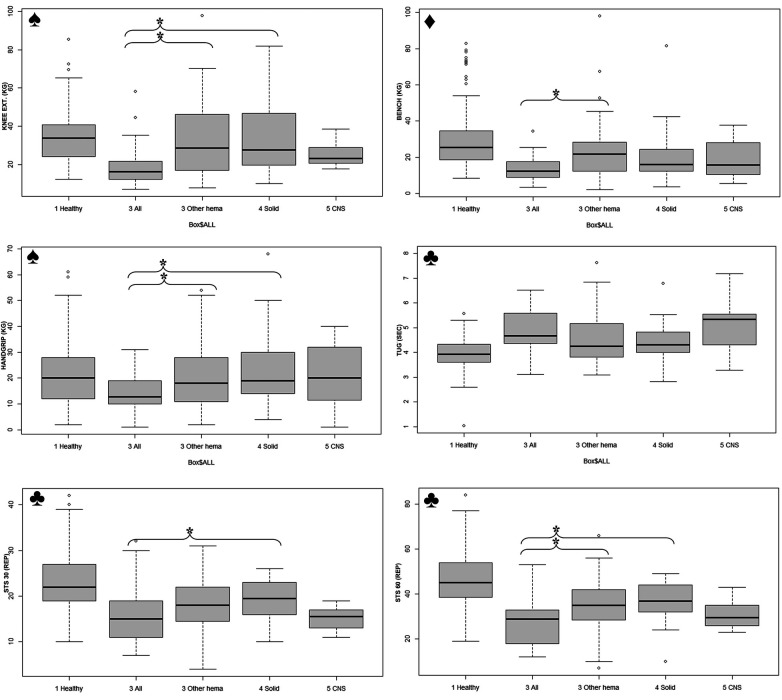
Differences in muscle fitness and physical function between diagnostic groups and community controls. In this figure, hematological cancers are divided into two groups of children with ALL and all other hematological cancers (Other hema). ♣ = Significant deficits across all diagnostic groups compared to community controls. ♤ = Significant deficits in children with hematologic cancers (ALL + other hematologic cancers) compared to community controls. ♦ = Significant deficits in children with ALL, other hematologic cancers, and extracranial solid tumors compared to community controls (no significant deficits observed for CNS tumors). * = Significant deficit between diagnostic subgroups within the bracket.

Significant differences in muscle fitness and physical function were observed between diagnostic groups across multiple outcome measures, as outlined in Figure 2.

Across diagnostic groups, children with hematological cancers displayed lower muscle fitness than those with solid tumors, particularly in knee extension, handgrip, and sit-to-stand performance (both 30 and 60 s). The largest deficits were seen in children with ALL (Figure 2).

### 3.4 Association between muscle fitness and time from treatment initiation in children with cancer

When exploring the association with treatment duration, upper body strength (bench press and handgrip strength) declined significantly over time during the first month of therapy, independent of age, sex, and diagnosis (Table 4). In contrast, treatment duration was not significantly associated with any of the other outcome measures. Additionally, no significant associations were observed for any outcome measure when analyses were conducted within specific diagnostic groups (Table 4).

**Table 4:** Associations of muscle fitness and physical function with time since initiation of cancer treatment.

Associations of knee extension, bench press, hand grip strength, countermovement jump, timed up and go test, sit-to-stand (30 and 60 s) with time from treatment initiation									
	Pan-cancer			Hematologic cancers		Solid tumors		CNS tumors	
Outcome	Unadjusted mean diff [95% CI]	Age+Sex + time from treatment initiation mean diff [95% CI]	Age+Sex + diagnosis + time from treatment initiation mean diff [95% CI]	Unadjusted mean diff [95% CI]	Age+ Sex + time from treatment initiation mean diff [95% CI]	Unadjusted mean diff [95% CI]	Age+ Sex + time from treatment initiation mean diff [95% CI]	Unadjusted mean diff [95% CI]	Age+ Sex + time from treatment initiation mean diff [95% CI]
Knee extension	−0.56 [−1.07 to −0.04]*	−0.30 [−0.65 to 0.06]	−0.34 [−0.69 to 0.01]	−0.54 [−1.14 to 0.05]	−0.32 [−0.73 to 0.08]	−0.81 [−2.08 to 0.47]	−0.67 [−1.67 to 0.33]	−0.40 [−1.66 to 0.85]	0.05 [−0.40 to 0.50]
Bench press (kg)	−0.66 [−1.21 to −0.10]*	−0.47 [−0.94 to −0.01]*	−0.53 [−1.01 to −0.05]*	−0.61 [−1.38 to 0.17]	−0.54 [−1.19 to 0.10]	−0.83 [−1.90 to 0.23]	−0.66 [−1.93 to 0.61]	−0.68 [−2.13 to 0.77]	−0.12 [−0.70 to 0.47]
Handgrip (kg)	−0.39 [−0.70 to −0.07]*	−0.21 [−0.40 to −0.03]*	−0.24 [−0.42 to −0.06]*	−0.35 [−0.70 to 0.01]	−0.18 [−0.39 to 0.02]	−0.57 [−1.35 to 0.22]	−0.33 [−0.79 to 0.13]	−0.65 [−1.92 to 0.62]	−0.28 [−1.01 to 0.44]
Counter-Movement jump	0.09 [−0.35 to 0.53]	−0.09 [−0.30 to 0.48]	−0.02 [−0.46 to 0.42]	–	–	–	–	–	–
Sit-to-stand 30s (repetitions)	−0.06 [−0.23 to 0.10]	−0.09 [−0.25 to 0.07]	−0.10 [−0.26 to 0.06]	−0.08 [−0.30 to 0.14]	−0.11 [−0.32 to 0.11]	0.04 [−0.28 to 0.36]	0.05 [−0.33 to 0.43]	−0.18 [−0.43 to 0.07]	−0.19 [−0.49 to 0.11]
Sit-to-stand 60s (repetitions)	−0.09 [−0.42 to 0.23]	−0.13 [−0.46 to 0.20]	−0.16 [−0.49 to 0.18]	−0.13 [−0.56 to 0.31]	−0.17 [−0.60 to 0.26]	0.05 [−0.64 to 0.74]	0.01 [−0.80 to 0.83]	−0.27 [−0.90 to 0.36]	−0.34 [−0.98 to 0.30]
Timed Up and Go (sec)	0.06 [−0.02 to 0.13]	0.07 [−0.01 to 0.14]	0.06 [−0.02 to 0.13]	0.05 [0.00 to 0.10]	0.05 [0.00 to 0.10]*	0.04 [−0.33 to 0.41]	0.18 [−0.27 to 0.63]	0.06 [−0.04 to 0.15]	0.05 [−0.08 to 0.19]

Associations of muscle fitness and physical function with time since treatment initiation across diagnostic groups.

* = *p* < 0.05.

## 4 Discussion

In this cross-sectional study of children assessed early after cancer treatment initiation, we observed substantial deficits in muscle fitness and functional performance compared with community controls, indicating that impairment is already pronounced at the point of diagnosis/early therapy. However, these impairments were not uniform across all children with cancer.

Diagnostic subgroup analyses revealed that children with ALL demonstrate the most pronounced and consistent reductions across all physical outcomes, suggesting they are particularly vulnerable to early deconditioning. In contrast, children with extracranial solid tumors and CNS tumors show either minimal or inconsistent differences relative to community controls, with isolated deficits in functional tests like TUG and sit-to-stand.

Children with ALL typically undergo the most prolonged and systemically intensive treatment regimens among pediatric cancers, especially during the induction phase, which involves high-dose corticosteroids, vincristine, asparaginase, anthracyclines and sometimes intrathecal methotrexate (3, 25). Both the systemic nature of the disease and the highly toxic induction regimens likely contribute to the substantial physiological stress, fatigue, and neuromuscular toxicity, providing a plausible explanation for why children with ALL present with the most marked deficits already early in the treatment trajectory, although impairments were evident across diagnostic groups.

Overall, the physical deficits described in this cross-sectional study are in line with the current body of evidence, which presents impairments in knee extension strength (12, 26), handgrip strength (12, 27–30), the Timed Up and Go test (27, 31, 32), and the 30-second sit-to-stand test (30) within the first month following cancer diagnosis. This cross-sectional study represents the largest — to date — study comparing individuals diagnosed with cancer with community controls of similar age and sex on outcomes of muscle fitness and physical function, and therefore adds an important factor for improving the precision of these results, albeit heterogeneity persists, as the current study uses a pan-cancer population. Being aware of this heterogeneity, we have reported outcome measures across diagnosis, and have further reported analyses adjusting for age, sex, and the potential heterogeneity of assessment time since treatment initiation. Together, these results represent a step forward strengthening the evidence base supporting early screening and assessment of physical function in children and adolescents with cancer.

These findings are further supported by our previous systematic review and meta-analysis, which synthesized physical capacity outcomes during the first months of cancer treatment and similarly demonstrated impairments in overall physical capacity, including muscle fitness, within the first month following diagnosis (2). Although heterogeneity across studies reduces the certainty of evidence, inflicted by using a pan-cancer population and small study populations, the directions of these impairments are consistent.

The regression analyses indicated that time-related declines were observed in upper-body strength (bench press and handgrip) during the first month of therapy, independent of age, sex, and diagnosis, whereas lower-body strength and physical function outcomes indicated no time-related declines. While this pattern may initially appear inconsistent with literature describing early global sarcopenia during intensive therapy (25, 33), and with studies on vincristine-induced peripheral neuropathy showing early onset in distal lower-limb nerves (34) with associated gait impairments (35, 36) these findings should be interpreted cautiously. Our analyses are cross-sectional and were not designed or powered to detect within-patient changes, especially given that most children were assessed within a median 9 days after treatment initiation. Therefore, these apparent discrepancies are more likely to reflect methodological constraints rather than indicating that deconditioning selectively affects upper-body musculature in the early phase. Rather, when considered alongside the large deficits observed across all outcomes in children with cancer, the data collectively support that substantial impairment is already present at diagnosis and may continue to accumulate rapidly during the earliest phase of treatment.

### 4.1 Strengths and limitations

Despite the inherent challenges of conducting physical assessments early in treatment, a relatively high proportion of children with cancer completed at least one outcome measure, supporting the representativeness of our sample for children in the acute treatment phase. Physical assessment in the early stages of treatment is inherently challenging due to logistical constraints, fluctuating symptoms, and variability in mood and treatment-related deficits (31, 37, 38). Additionally, parents often report acute stress during this period, further complicating the assessment of general physical function (39). The complexities of conducting physical assessments, and physical exercise, within the early stages of cancer in the current study sample have been described elsewhere (37). However, we cannot exclude the possibility of selection bias, as 22% of eligible participants were not included. Participation in early physical assessments may have been influenced by clinical status, potentially favoring children who were sufficiently stable to complete testing.

The reasons for non-participation, as presented in the study flowchart, primarily reflect clinical and contextual factors during the early phase of treatment, including inability to engage in non-treatment activities. Although we were not permitted to collect or report demographic or clinical data from non-participants, findings from a related study evaluating the feasibility of participating in exercise interventions within the same population, suggest that non-participation and incomplete assessments are largely driven by treatment burden and acute symptoms (e.g., pain, fatigue, and nausea), rather than specific demographic or diagnostic characteristics (37). This may indicate that our sample represents children who were clinically stable enough to participate in physical assessments, and that the observed impairments could therefore be underestimated.

A limitation of this study is the variation in assessment timing relative to the start of cancer treatment, which introduces heterogeneity in treatment-related effects on muscle fitness.

This study is the first to report measures of muscle power using the countermovement jump. However, this assessment was only available at one site, resulting in a limited sample size (*n* = 24 children with cancer) and a potential risk of selection bias. Nevertheless, the countermovement jump—reflecting explosive strength and rate of force development—can be considered a functional surrogate for muscle quality. Muscle quality is of particular interest in children and adolescents with cancer, as force or power per unit of muscle mass is directly related to key aspects of physical function, including balance, and the ability to walk, run, and jump (1).

Subgroup analyses, particularly for children with CNS tumors, were based on small sample sizes, resulting in wide confidence intervals and limited precision. While effect estimates are reported, these findings should be interpreted with caution.

Finally, we did not account for acute treatment-related symptoms such as pain, fatigue, or nausea, which may influence physical performance and contribute to variability in the observed outcomes.

### 4.2 Implications for practice and research

While overall impairments were most pronounced in children with ALL within the first month after treatment initiation, time-related declines were not diagnosis-specific. Nevertheless, these findings highlight the importance of early screening and targeted strategies to preserve muscle fitness during treatment. More broadly, early muscle loss and sarcopenia are common in pediatric oncology (25), and in oncology in general, and have been associated with increased risk of invasive fungal infections and prolonged hospital stays, with mixed evidence regarding mortality (33). Taken together, these findings underscore the importance of pragmatic, early screening approaches combined with targeted exercise and supportive care to identify children at risk and mitigate potential complications.

Because the present study is cross-sectional, the observed differences across time points reflect between-participant variation rather than within-participant change. Thus, while our findings suggest that physical deconditioning is already evident early after diagnosis—and likely begins even before treatment initiation—precise determination of its onset and progression would require a longitudinal data with repeated within-participant assessments. However, given the substantial logistical and physical burden of early cancer treatment, such intensive monitoring is rarely feasible. In this context, conducting a single early assessment within the first month may represent a clinically meaningful and practical approach for identifying children at risk and initiating timely interventions.

## 5 Conclusion

Children and adolescents with newly diagnosed cancer show clinically relevant impairments in physical function and muscle fitness—including muscle strength, muscle power, and muscle endurance—within the first month of treatment compared with community controls, with the largest deficits in acute lymphoblastic leukemia. In exploratory analyses, upper-body strength (bench press and handgrip) declined with increasing days from treatment initiation, whereas other outcomes showed no clear time-related change; these findings should be interpreted cautiously given the cross-sectional design. Together, the results support early screening and timely, targeted rehabilitation to mitigate early deconditioning.

## Data Availability

The datasets generated and analyzed during the current study are not publicly available due to Danish and EU personal data protection legislation, but de-identified data may be made available from the corresponding author upon reasonable request and with appropriate approvals.
